# Development and evaluation of an online education tool on attention deficit hyperactivity disorder for general practitioners: the important contribution of co-production

**DOI:** 10.1186/s12875-020-01289-5

**Published:** 2020-11-01

**Authors:** B. French, D. Daley, E. Perez Vallejos, K. Sayal, C. L. Hall

**Affiliations:** 1 Division of Psychiatry & Applied Psychology, University of Nottingham, Nottingham, UK; 2 Centre for ADHD and Neurodevelopmental Disorders Across the Lifespan (CANDAL), Institute of Mental Health, University of Nottingham, Nottingham, UK; 3 NIHR Nottingham Biomedical Research Centre for Mental Health and Technology, Nottingham, UK

**Keywords:** General practitioners, ADHD, Online intervention

## Abstract

**Background:**

Attention deficit hyperactivity disorder (ADHD) is underdiagnosed in the UK and the assessment and diagnosis pathway often involves a general practitioner (GP) referral to secondary care services. GPs’ levels of knowledge and understanding about ADHD is often a significant barrier in patients accessing care. The development of an online education resource could improve GPs knowledge of ADHD and optimise appropriate referrals. Involving end-users in co-creating interventions may enhance their clinical utility and impact routine clinical practice. However, there is limited published evidence describing how to meaningfully involve stakeholders in both the design and development components of co-production.

**Method:**

We report a step wise, co-production approach towards developing an online ADHD education intervention for GPs. Preparatory work highlighted the relevant topics to be included in the intervention, from which educational videos were then developed. Workshops were then conducted with GPs, leading to further refinement of the video content and subsequently the final intervention. A pilot usability study (*n* = 10 GPs) was then conducted to assess the intervention’s acceptability, feasibility and accessibility.

**Results:**

The development of the online intervention was greatly facilitated by the involvement of GPs. Having a co-production development process ensured the consistent adaptation of the intervention to meet GPs’ needs. The usability study showed that the content of the intervention was suitable, easily accessible, engaging and delivered at an acceptable level of intensity, validating the development approach taken.

**Conclusion:**

While further studies are needed to evaluate the efficacy of the developed intervention, preliminary findings demonstrated that it was acceptable and well received. The importance of co-development was highlighted in developing an intervention that addresses specific needs for GPs. This development approach may be useful for other researchers and developers of clinical interventions.

## Background

Attention deficit hyperactivity disorder (ADHD) is a neurodevelopmental disorder affecting 3–5% [1, 2] of children, with symptoms often continuing into adulthood. In the UK, ADHD is widely underdiagnosed and under-treated with 0.73% of children receiving ADHD medication [3]. The symptoms experienced by children with ADHD can lead to considerable behavioural and cognitive impairment [4, 5] affecting many aspects of their lives. In adulthood, the risk associated with undiagnosed and untreated ADHD, such as higher risk of suicide or loss of work can have strong economic and social burdens [6]. Gaining a diagnosis of ADHD is important for access to appropriate treatment. The diagnosis pathway for ADHD involves multiple stakeholders such as parents, teachers and healthcare professionals. Given that ADHD symptoms need to be present in two environments in order to make an ADHD diagnosis, school involvement is critical for informing diagnostic decisions. GPs have a key role in ADHD diagnosis in that they act as gatekeepers to secondary care services where diagnosis and treatment of ADHD takes place. GPs do not always readily recognise ADHD symptoms or impairment; many report low confidence and limited knowledge on the condition [7]. This is a key barrier for children at risk of ADHD accessing care. Despite this, currently there are few evidence-based interventions aimed at improving GPs knowledge and confidence of ADHD. The development of interventions targeted at increasing their knowledge and confidence is therefore essential.

To address this issue, we developed an online intervention called “Understanding ADHD in primary care” which aimed to increase GPs’ understanding and awareness of ADHD. By increasing ADHD awareness and knowledge this intervention aimed to increase support for ADHD in primary care, and facilitate identification and appropriate referral. Current ongoing research indicates that this tool may be effective in improving ADHD knowledge and subsequent referrals [8]. Given the potential success of this intervention, it is likely that this approach, utilising an online education resource, may be adapted and used to improve GPs’ knowledge of other mental health conditions.

Healthcare professionals’ use of online training has significantly increased over the last two decades [9, 10], with a US study reporting an increase of physicians taking part in online learning activities from 605,410 to 4,365,014 between 2002 and 2008 alone [9]. An online education resource offers many advantages for all types of healthcare professionals, including GPs. It can be easily accessible at times that work around GPs busy schedules and from most locations providing there is adequate broadband or mobile data available. Accessing resources online is particularly beneficial for those in remote areas. A recent literature review demonstrated that online training can significantly improve GPs knowledge and practice [11]. However, in order to promote uptake of these interventions in routine practice it is important that the developed intervention meets the needs of the end-user and is deemed feasible, and acceptable. Interventions tailored to address identified barriers have been shown to improve professional practice [12]. Co-produced research offers the opportunity to improve this. Although co-production is becoming a familiar term among healthcare researchers due to the opportunities for innovation and service improvement it provides [13], little is known about how to achieve the positive outcomes derived from co-production and the mechanisms/processes involved in co-production activities. Value co-production occurs in particular when users (i.e., GPs, patients, carers) are able to personalise their experiences and influence specific research tasks and outputs. This process requires active collaboration by users and researchers to create value. Embedding co-production activities into research is a way to promote responsible innovation and to ensure that the research outputs are relevant, engaging and desirable for end-users [14, 15].

Researchers acknowledge that co-produced research may be challenging, involving a complex balance of different expectations, goals and experience, however studies have also found that researchers learn a lot from involving end-users in their studies [16]. The majority of papers do not describe their methodological development process beyond publishing their protocol [17]. However, sharing experiences of the process of co-producing interventions provides the opportunity for greater critical appraisal of interventions and may facilitate knowledge exchange.

The aim of this paper is to report the methodological development of the online education resource. To ensure that the intervention met the needs of the end-users (GPs) the intervention was co-produced by GPs and underwent three iterative steps, with input from GPs at each stage. To achieve this, the research team worked collaboratively alongside an external team which specialises in health e-learning resources (HELM - Health E-Learning and Media). HELM applies a specific development program involving co-production between academics and stakeholders. Here, we detail the steps to developing the intervention alongside a small pilot study to test the usability and acceptability of the intervention.

## Method

**Fig. 1:**
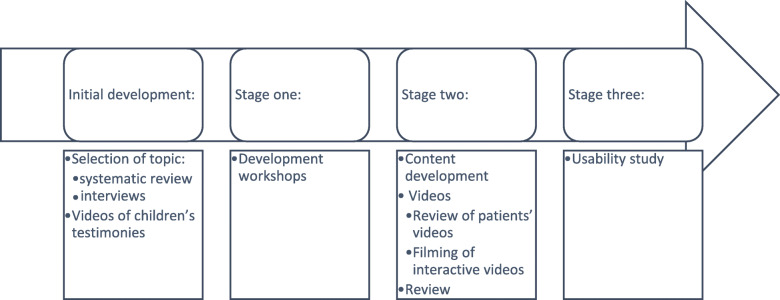
Flow chart of the development process

The development of the online intervention “Understanding ADHD in primary care” was conducted over 8 months and included multiple steps, with input from various stakeholders at each point. A brief overview of the development process is shown in Fig. 1.

### Initial development

#### Selection of topics

In order to guide the development of the content of the intervention, specific ADHD topics were selected that targeted previously identified unmet needs of GPs when managing ADHD.

The selection of these topics was guided by the findings of previously conducted studies in this area [7, 18]. These studies highlighted specific barriers in GPs understanding of ADHD, a summary of the main barriers are presented in Table 1.

**Table 1:** Summary of themes relating to awareness and understanding of ADHD in primary care

Systematic review (French et al, 2018)	Pilot study (French, unpublished thesis chapter)	Semi-structured interviews (French et al, 2020)
Need for education	Negative connotation of ADHD	Lack of identification in primary care
Misconceptions and stigmas	Parenting	Lack of clear diagnostic pathway and services
Constraints with recognition, management and treatment	Social background	GPs’ knowledge of ADHD and misconceptions
Multidisciplinary approach	Lack of experience/knowledge	Difficult communication between multiple stakeholders
	Diagnosis / consultation procedure	Impact of diagnosis and the risks linked to no diagnosis

The collective findings from these three studies can be broadly categorised into two main concepts – [1] issues around knowledge and [2] issues around the complexity of the diagnostic process. As such, these two concepts were the focus of the intervention. Based on this, the team developed videos with the aim of capturing the lived experiences of patients around these two themes

#### Videos

Two sets of videos were used for this intervention. One set of videos filmed five adults (male and female) talking about their lived experiences and the daily impact of ADHD. The second set of videos filmed eight children with ADHD and their parents talking about their experiences. The video participants were recruited from support or charity groups and signed consent (or parental consent in the case of the children) for the use of their videos. The videos were co-designed by a consultant psychologist and the research team. By giving accounts of service users, the videos aimed to be a central part of the intervention in explaining the daily impacts of ADHD.

Two lead researchers (BF and DD) reviewed the footage and condensed the content down to two 5-min video clips which contained the most important information pivotal to the themes shown in Table 1.

Following the video development, an initial outline of the intervention emerged which centred on the video discussions pertinent to the two main barriers (lack of knowledge and complexity of diagnosis).

As such, the proposed content for the online intervention focussed on:

1. Understanding the different roles held by different stakeholders
2. Understanding the role of the GP
3. Understanding the diagnosis pathway
4. Improving general knowledge of ADHD
5. Dispelling common myths on ADHD
6. Socioeconomic status (SES), parenting and the child’s behaviour in the consultation
7. Understanding and challenging common negative conations of ADHD
8. Benefits of receiving an assessment and/or diagnosis
9. Risks of untreated ADHD

#### Development process

The online intervention was developed in partnership with the HELM (Health E-Learning and Media) team from the University of Nottingham, UK. The HELM team specialises in media-based educational materials and intervention in health and were chosen for the development of the online resource due to their expertise in the area. The HELM development process has specific stages to ensure the most optimal final product and learning outcomes, which has established efficacy.

Stage one - a workshop is set up with service users or the population of interest (in our case GPs) in order to develop a targeted resource that is appealing and accessible to its users [19]. This workshop creates a set of storyboards that informs the content of the resource. Specifications for the resource are then developed (by BF in this project) including but not limited to written content, exercises, interactive activities and assessment.

Stage two - A peer review process follows whereby the proposed content is reviewed by an expert on the topic that has not been involved in the development. The creation phase then starts and is solely conducted by the HELM team. Upon completion of the online resource, another review process is conducted where a reviewer and the team assessed the final product before dissemination.

The development process that we followed, paralleled this process while also incorporating an additional third Stage – a usability study to assess the intervention. Here, we outline each of these three stages.

### Stage 1: development workshops

To further develop the intervention and in line with the HELM process, workshops were conducted with 15 GPs and two other healthcare professionals.

In a variation from the HELM process, we held two separate workshops to enable ideas from the first workshop to be presented to the second workshop members for further development and validation of the original concepts.

#### Workshop members

The first workshop included the lead researcher (BF) and 11 GPs and the second workshop four GPs and two secondary care professionals specialising in ADHD assessment - one from child services and one from adult services. The two secondary care specialists were included to gain more specific input to the content of the resource.

The workshops lasted 3 h and participants were compensated for their time. After a brief presentation of the research project and the HELM team, the participants were split into three groups and asked to work on storyboards for the resource. They were specifically asked to think about the format and appearance of the resource rather than specific content. Examples of online resources were presented in order to facilitate ideas. The three groups then presented their storyboards to the whole group.

The second workshop was run using a similar format however, a review element was added due to the smaller numbers. In addition to the storyboards, the participants in this workshop were presented with a summary of the suggestions from the first workshop and asked to review these suggestions. The participants were split into two groups, each tasked with creating a storyboard. These storyboards reinforced specific content suggested in the first workshop but also brought out some new ideas. For instance, the participants felt that the videos developed originally were not useful and suggested more targeted, shorter videos focusing on symptoms.

Table 2 presents the main ideas that emerged from the workshops.

**Table 2:** Main suggestions presented at the workshops

Suggestions from the workshops	
Making two short online resources, one specific to ADHD (Symptoms, epidemiology…) and one specific to the GPs role in diagnosis and treatment	
Including information on the benefits of diagnosis, what can happen without treatment (information on prison statistics, substance abuse, suicidality...)	
Shorter videos of patients focusing on symptoms	
Adding expert videos on symptomatology and secondary care pathways. What happens after a referral	
Separating clearly child and adult pathways, having a child specific module and an adult specific module	
Adding an assessment at the end in the form of a multiple choice questionnaire	
Including information on comorbidities in the form of a diagram	
Including information on ADHD at different ages	
Adding access to resources for management and for patients’ information (Parenting websites, ADHD support groups, charities…)	
Comprehensive information on treatments	
What is the role of the GP?	
Drag and drop activities on myth versus facts	
Including an example of a consultation	
Information on local pathways	

### Stage two: content development and review

#### Development group

The development group involved seven stakeholders with specific ADHD expertise. It included the lead research team and additional group members. The research team included: the lead researcher (BF) and two supervisors with ADHD-related expertise (DD, KS). The group also included two healthcare professionals working with adults and children with ADHD, one GP who was diagnosed with ADHD and one GP who had carried out research on ADHD as part of their PhD. The role of this group was to act as a form of steering committee, overseeing all aspects from the developing intervention and making decisions on final content. Some of the group members facilitated and attended the workshops (BF, AG, JK) while others’ roles were more focused on reviewing the content.

Two members of the development group (BF, DD) synthesised the information from the workshop and developed a draft intervention. The group were mindful to include different activities within the intervention to keep the content entertaining and engaging. Examples of activities include drag and drop games, questionnaires on myths about ADHD and animated pictures of brain correlates. The draft was reviewed by the rest of the development group and sent to HELM to create the intervention. A summary of the changes made based on the workshop is provided below.

#### Integration of recommendations

Most recommendations from the workshop were integrated into the online resource. However, some recommendations had to be discarded. Table 3 presents these suggestions and the research team’s rationale for or against implementation.

**Table 3:** Implementation of the main suggestions presented at the workshops

Suggestions from the workshops	Implementation
Making two short online resources, one specific to ADHD (Symptoms, epidemiology…) and one specific to the GPs role in diagnosis and treatment	Instead of one module, we separated the content into two modules: “Understanding ADHD” and “The role of the GP in the diagnosis and treatment process”
Including information on the benefits of diagnosis, what can happen without treatment (information on prison statistics, substance abuse, suicidality...)	A page on the risks of undiagnosed and untreated ADHD was added with research statistic accentuating the importance of early intervention
Shorter videos of patients focusing on symptoms	The videos were changed to make them symptom specific. The patients’ testimonies were restructured and six shorter videos were developed focusing on features of hyperactivity, inattention and impulsivity in adults and in children
Adding expert videos on symptomology and secondary care pathways. What happens after a referral	Expert videos were added. Four ADHD experts were filmed to give a specialist opinion on specific topics. A GP with a diagnosis of ADHD, related her lived experience of being both a GP and a patient with ADHD. A lead researcher on ADHD (DD), discussed strategies to help support ADHD patients during the diagnosis process and non-pharmacological approaches. An advanced nurse practitioner and a consultant psychologist, explained the secondary care process following referral
Adding an assessment at the end in the form of a multiple choice questionnaire	A quick assessment on ADHD knowledge was added at the beginning and at the end of the modules to assess any changes in participants’ knowledge
Including information on comorbidities in the form of a diagram	The diagram idea was added to the page on comorbidities in order to improve understanding of overlapping conditions
Including information of ADHD at different ages	An infographic was created to show the development of ADHD symptoms through the ages
Adding access to resources for management and for patients’ information (Parenting websites, ADHD support groups, charities…)	A toolkit was created at the end of the module where many resources on management, support groups, screening etc. can be found
Comprehensive information on treatments	The pages on treatment were expanded to include pharmacological and non-pharmacological treatments with details on the specific types of medications
What is the role of the GP?	The first page of the second module included a concise summary of what the role of the GP is exactly, and what it isn’t
Drag and drop activities on myth versus facts	An interactive drag and drop activity was created to address typical misconceptions about ADHD
	**Suggestions that could not be implemented**
Including an example of a consultation	GPs suggested including a video of a mock consultation. While it would have been very interesting to implement this idea, adding an extra 10 min of videos to encompass a whole consultation felt too lengthy. Furthermore, identifying ADHD in patients is very different depending on many factors such as the type of ADHD, the age or the gender and it was felt that we couldn’t represent it all accurately in one mock consultation
Separating clearly child and adult pathways, having a child specific module and an adult specific module	This suggestion was addressed to an extent by clearly specifying the differences in child and adult pathways when relevant. However, it seemed too repetitive to create separate modules for each as a lot of the information overlapped
Information on local pathways	Information on local services and pathways was unanimously the one piece of information GPs wanted to receive the most. However, it is impossible to know the different pathways in each British locality as firstly, there are so many and secondly, services are constantly changing in response to commissioning decisions. However, a statement was added to explain that local services information needed to be sought by the GPs in order to offer best access to care

#### Reviews

A thorough review process was implemented throughout the development process. The modified online intervention (see Table 3) that developed from the workshops and development group review then underwent three further reviews:

- A GP first reviewed the content to ensure it was appropriately targeted to GPs. The content specifications were also sent to a reviewer (KS) who had not taken part in the content development. Additionally, it was proof read by a professional proof reader.
- Following the online development, the final resource was produced. The lead researcher (BF) reviewed the content to ensure the resource was developed according to the original specifications. The resource was then sent to an external reviewer to assess time, accessibility, content and format.
- Finally, the resource was reviewed by the Royal College of General Practitioners (RCGP) in order to receive accreditation. This accreditation was suggested in the co-production process as an increase incentive for uptake in the intervention. Upon seeing the final version, a few details had to be addressed in order for the accreditation to be granted. This feedback was minor, easily addressed and accreditation was received in July 2019.

#### The developed intervention: “Understanding of ADHD in primary care”

The developed intervention was called “understanding of ADHD in primary care” and was delivered on an open source learning management system from a University of Nottingham server. The complete online intervention consists of two 25-min modules undertaken sequentially. The two modules follow the same format of having text on the left hand side of the screen and interactive activities on the right. The activities varied and included patient testimonies, drag and drop games, videos and pictures.

- Module 1: “Understanding Attention Deficit Hyperactivity Disorder” introduces the many aspects of ADHD. After a brief description of ADHD epidemiology and neuroscience, the core three symptoms are discussed with real life settings examples. Other symptoms, common misconceptions and key impacts on children and adults are also discussed. Finally, comorbidities and risks associated with ADHD are presented.
- Module 2: “The role of General Practitioners in ADHD diagnosis and management” introduces in more detail the GP’s role in the ADHD diagnosis and treatment pathways. Clarifying the gatekeeping role held by GPs and the pathway to care in the UK, this module also expends on identification of ADHD, treatment options and the effect of gaining better ADHD knowledge on practice. Finally, an “ADHD toolkit” included with various downloadable forms such as screening tools, strategies or useful websites.

This resource can be found on: www.adhdinfo.org.uk

### Stage three – usability study

To determine the usability of the intervention, a pilot study was conducted with 10 GPs. The aims of the pilot study were to assess the intervention usability, to ensure that the intervention ran in a timely manner and that no technical errors occurred.

#### Participants

GPs who had registered consent to contact after taking part in previous interviews and in the development workshops were contacted by a member of the research team to review the usability of the online resource. Fifteen GPs were approached and ten GPs (4 females) completed the intervention study (66% response rate). Seven GPs had taken part in both interviews and workshops while three only took part in the interviews.

#### Measures

##### Usability questionnaire

A usability questionnaire was developed, containing 29 questions assessing key usability criteria such as learnability, efficiency and memorability. Question type varied from forced choice questions (“I will use this tool in the future”-agree, disagree, unsure) and free text questions (“Were any parts of the tool not helpful?”).

#### Procedure

GPs who had given consent to be contacted after taking part in previous studies were emailed details about the study and sent links to an online information sheet and consent form to complete in order to take part. Upon receiving consent, GPs who agreed to take part were then sent a link to the intervention with embedded outcome measures. While some participants had taken part in the initial development workshops, none of them were familiar with the final online intervention. GPs were advised to set aside 90 min to complete the study in one go. Although it was not encouraged, participants were able to stop the study at any point and come back to it at a later point. The usability questionnaire was completed immediately after finishing the intervention as part of a suite of outcome measures (further details in other manuscript, 8).

Upon completing all questions, participants were given an inconvenience allowance and a Continuing Professional Development (CPD) certificate from the RCGP.

Descriptive analyses were used to summarise the findings from this study.

## Results

### Usability and acceptability

Ten GPs took part in the usability study. Nine were aged between 25 and 35 years and one between 36 and 45 years. Years of practice since qualifying as a GP ranged from 10 months to 11 years (mean: 6y 7 m).

The completion time (including the questionnaires and intervention) ranged from 45 to 72 min although it was not possible to assess the response time of two participants as they did not complete the intervention in one sitting.

Results from the usability questionnaire are presented below. Participants were asked to rate some questions on a scale of 1 to 10 (Table 4) and others if they agreed or disagreed with specific statements. Free text questions on their overall interaction with the resource were also included.

**Table 4:** Usability and acceptability evaluation on a scale of 1–10 (1: not at all and 10: a lot). Table values represent the number of responses for each scale point

Scores	Total mean	SD	Range
How confident are you in your knowledge of ADHD			
Pre intervention	**6.2**	**1.48**	**3–8**
Post intervention	**7.9**	**0.94**	**6–9**
How useful did you find the information in this programme?	**9.2**	**0.87**	**8–10**
Did you like using the tool?	**8.5**	**0.67**	**8–10**
Do you feel the tool impacted your knowledge on ADHD?	**8.5**	**0.92**	**6–10**
How likely is this information going to inform your practise?	**8.7**	**0.64**	**8–10**
Do you believe the content was relevant to your practice?	**9.2**	**0.87**	**8–10**

The participants reported a high degree of satisfaction with the content and layout of the online intervention. All participants were able to navigate through the resource easily, and only one suggestion was made to improve navigation. The wording and presentation of the content was well received, participants reported the content to be clear, interactive and easy to follow. All participants also felt that the resource was useful, increased their knowledge and was relevant to their practice and confirmed they would recommend the resource. While a few suggestions for improvement were made, the feedback was overall strongly positive.

### Positive feedback

All participants agreed that they will definitely recommend the resource and most felt that no parts of the resource were unhelpful or that anything was missing from the content. The additional comment section contained mainly positive comments where participants principally highlighted that they liked the interactivity and the structure of the resource. The participants especially liked the videos used to reinforce their learning.

*“Great resource, videos help to give a true account” (P4)*

*“Good mix of bullet point text and short videos. Interplay between the two helped reinforce points” (P10)*

While most agreed that the resource was the right length, a couple of participants that suggested the resource might be too long but acknowledged that despite feeling that it might be a bit lengthy, they wouldn’t know which part to cut out.

*“It was (too long), hard to decide what was the least useful. All useful stuff” (P5)*

### Suggestions for improvement

Only a few suggestions for improvement were made relating to the length and format of the intervention, the content, and navigation.

#### Length and format

While participants were mostly satisfied with the length of the intervention, one participant highlighted that it was important to advise participants of how long it will take beforehand. Another participant suggested highlighting the key points from each slide to make it quicker, with take home messages in bold.

#### Content

Two participants suggested improvement of content. One suggested inputting a bit more information on the difference between autism and ADHD. The other participant suggested including more information on treatments, management and monitoring.

#### Navigation

Finally, the last suggestion for improvement was in relation to the navigation of the resource. The participant suggested that the two modules would flow better in one module rather than two separate modules.

Questions on the usefulness of the resource in practice were also asked to ensure that the content did help to increase awareness of ADHD. All participants agreed that the resource will help them identify ADHD patients better, all believed that they will retain the knowledge acquired from the intervention and that it impacted on their attitude towards ADHD and ADHD patients.

## Discussion

The objective of this exercise was to develop a robust and feasible online psycho-education intervention for GPs. In following a systematic step-wise development process and with the aim of co-developing a psychoeducation tool to improve GPs’ knowledge of ADHD, we created an online intervention involving GPs at each stage of development. The resulting online tool was tested for its usability and considered to be highly functional and acceptable. We provide further reflections on the online delivery of education interventions and the co-production of the intervention, highlighting the opportunites and challenges associated with these two (online and co-production) factors.

The online format of the intervention offered many advantages, which are likely to also be attractive to other healthcare professionals. The benefit of having an online resource that is freely available means that healthcare professionals can access it in their own time and from anywhere. As it is also easily accessible on smartphones, firewall restrictions from work desktop (such as the ones implemented by the NHS) were easily bypassed. It is therefore hoped that the resource can be used as a support tool as well as an education tool. By including downloadable files, GPs can refer back to this resource and extract documents to support their practice such as screening tools, support networks etc.

The final developed intervention had a few limitations. Completing the intervention takes approximately 45 min, in real-world practice time may be a barrier to completion. While it is accessible on smartphones the layout is not as intuitive on a small screen and much better impact of the videos and the interactive activities can be experienced on a computer. Finally, the intervention is specific to UK practice and while the first module on Understanding ADHD is internationally relevant, the second has many country-specific limitations as it aimed to clarify the role of the GP in the UK system.

The process followed by this study, demonstrated how co-production impacted the research as well as the researchers. Co-production allows for tailored interventions to be developed, specific to the users’ needs [13, 14] but also create a significant learning process for the researchers [15]. From the initial concept to the final product, the intervention changed in multiple ways. Through co-production a significant discrepancy was highlighted between the product originally envisioned and the product that GPs wanted. As a result of this, changes were made to the format, the content and the delivery of the intervention. The format of the intervention evolved greatly over the period of this project. For instance, while it was originally thought that the patients’ testimonies would hold a significant part of the intervention, the GPs feedback meant that it was considerably edited. The length of the intervention was also a contentious point with GPs preferring an intervention as short as possible, while incorporating all the necessary information. A compromise was reached with a 45 min’ online intervention. For similar reasons, the content of the intervention was continuously adjusted and evaluated over the length of the development process. It was important to find the right balance between enough information for them to learn and be engaged, as per the original research objectives, but not too much so they became bored or over-loaded. Finally, the mode of delivery was considered carefully. From early on, an online intervention felt the most suitable to meet GPs’ needs, as opposed to web based talks or workshops. Platforms of delivery were not significantly important, but accessibility was essential, being able to access the resource from an NHS computer or a smartphone a requirement. Furthermore, accreditation from a reliable source (RCGP) was also essential both in order to validate the resource itself and also to gain CPD accreditation points from taking part.

The online resource has been adopted by two primary GP training resources. Firstly, the RCGP, after accrediting this training programme for a year, has endorsed the online resource as part of their ongoing online continuous professional development (CPD) training. This is now part of the mental health toolkit part of the CPD online courses (https://www.rcgp.org.uk/clinical-and-research/resources/toolkits/mental-health-toolkit.aspx). Another online CPD site, Fourteenfish, which specialises in CPD training for GP trainees has endorsed the online resource and it now features on their online CPD course for trainees (https://www.fourteenfish.com/). Each of the two platforms has over 40,000 members.

The co-production aspect of the development, was the most informative part of this process. As stated above, the initial concept the researchers had planned was completely different to the final product. While this was at times frustrating as a lot of the original ideas and concepts had to be dropped, it was essential in developing a robust product that met the GPs’ needs. As healthcare professionals rely more and more on remote learning [8], it is important that this learning is tailored to their needs. Without this input and co-production process, the research team would have developed a product that met their beliefs about what GPs need but would not have engaged the GPs which would have been pointless. It was also very important to choose the right reviewers in the process. When the template was first scrutinised by academic members of the study team, their feedback sometimes differed from the GPs’ preferences. The GP reviewers understood the decisions made about the format, content and delivery of the intervention.

A few limitations to the process of development can also be highlighted. Firstly, seven of the 10 participants in the usability study had been members of the workshops. While the workshops produced six storyboards and the participants were not aware of which suggestions were going to be implemented, those seven participants all had an input at an early stage of the development process. Secondly, the participants from the workshop and from the usability study were all young self-referred motivated GPs. The perspective of older and more experienced GPs is therefore lacking. While recently qualified GPs are used to online training resources, more experienced GPs might have had very different opinions on the format, the content and the delivery of the intervention. As we know ADHD awareness has significantly increased in the last decade [20], it could be assumed that GPs who completed their training over 20 years ago might hold different knowledge and attitudes towards ADHD. Their standpoint would have therefore been very valuable in the development of the intervention. Finally, the usability study is restricted to the views of 10 GPs so generalisation needs therefore to be met with caution, however the intervention has been well accredited.

By developing a resource that meet GPs’ needs and increase their knowledge, this resource could also become a validated template for GP education, potentially being adapted towards other developmental disorders.

Following this careful methodology has allowed for a feasible and acceptable tool to be developed for GPs. A future randomised control trial is required to determine whether this influences practice over time, the quality of GP referrals for ADHD to secondary services and patients’ experience of services.

## Conclusion

The methodology followed to develop this online intervention resulted in the development of an acceptable and accessible learning tool for GPs. The step-wise and co-production process was essential in the success and positive outcomes of the intervention. Further research is warranted to assess the impact of this on practice, however this work has shown that interventions that are co-developed with the end-user may be more acceptable and feasible to implement.

## Data Availability

The datasets used and/or analysed during the current study are available from the corresponding author on reasonable request.

## Abbreviations

ADHD: Attention deficit hyperactivity disorder
CPD: Continuing professional development
GPs: General practitioners
HELM: Health E-Learning and Media
KADDS: Knowledge of Attention Deficit Disorders Scale
NHS: National Health Service
RCGP: Royal College of general practitioners
